# Associations between dietary intake and pancreatic disease: a Mendelian randomization study

**DOI:** 10.1097/JS9.0000000000003527

**Published:** 2025-09-22

**Authors:** Qi Zhang, Shuo Yu, Delong Gao, Jun Gong

**Affiliations:** a Department of Plastic and Cosmetic Surgery, Tongji Hospital, Tongji Medical College, Huazhong University of Science and Technology, Wuhan, Hubei, China; b Department of Biliary-Pancreatic Surgery, Tongji Hospital, Tongji Medical College, Huazhong University of Science and Technology, Wuhan, Hubei, China; c Department of Orthopaedic Surgery, Tongji Hospital, Tongji Medical College, Huazhong University of Science and Technology, Wuhan, Hubei, China

**Keywords:** dietary intake, Mendelian randomization, pancreatic cancer, pancreatitis

## Abstract

**Background:**

Pancreatic diseases, including acute pancreatitis, chronic pancreatitis, and pancreatic cancer, pose significant health challenges. The role of diet in these diseases is not well understood due to confounding factors in observational studies.

**Objective:**

This study aims to clarify the causal relationships between dietary intake and pancreatic diseases using Mendelian randomization (MR).

**Methods:**

A two-sample MR approach was employed, utilizing genetic data from the UK Biobank for dietary exposures and the FinnGen consortium for pancreatic disease outcomes. Genetic variants were selected as instrumental variables to assess the impact of 26 dietary components on acute and chronic pancreatitis, as well as pancreatic cancer. Meta-analyses were performed to validate findings across datasets, and multivariable MR (MVMR) analyses assessed the independent effects of dietary factors.

**Results:**

The analysis revealed that dried fruit intake was protective against both acute (OR = 0.396, *P* = 0.028) and chronic pancreatitis (OR = 0.289, *P* < 0.001). Conversely, red wine (OR = 1.559, *P* = 0.039) and bread (OR = 2.244, *P* = 0.044) were linked to increased acute pancreatitis risk. Pork was associated with chronic pancreatitis (OR = 3.652, *P* = 0.048), while oily fish intake correlated with a higher risk of pancreatic cancer (OR = 1.699, *P* = 0.046). Meta-analyses confirmed dried fruit protective association with acute pancreatitis. MVMR analyses indicated independent causal relationships between dried fruit and both acute and chronic pancreatitis.

**Conclusion:**

This study showed the protective effects of dried fruit and salad/raw vegetables against pancreatic diseases, while red wine, bread, and pork may elevate risk. Dietary modifications could serve as effective preventive strategies, warranting further exploration of underlying mechanisms.

## Introduction

Pancreatic diseases, mainly including acute pancreatitis, chronic pancreatitis, and pancreatic cancer, represent a critical public health challenge, characterized by high mortality rates, complex etiologies, and limited treatment efficacy^[1]^. Acute pancreatitis is characterized by sudden inflammation of the pancreas, often linked to gallstones or alcohol consumption, leading to severe abdominal pain and potential systemic complications^[2]^. Chronic pancreatitis, a progressive fibroinflammatory condition, results in irreversible damage to the pancreas, causing persistent pain, malabsorption, and diabetes^[3]^. Pancreatic cancer, known for its poor prognosis and late-stage detection, remains one of the deadliest cancers, with risk factors including smoking, obesity, and diabetes^[4]^. Despite advances in understanding genetic and environmental risk factors, the role of modifiable lifestyle factors, particularly diet, remains insufficiently elucidated. Identifying causal dietary drivers could significantly enhance preventive strategies, yet existing evidence from observational studies is inconsistent and confounded by methodological limitations.

It is well known that diet plays a crucial role in the development and progression of pancreatic diseases, influencing both risk and outcomes^[5]^. Observational epidemiology has long implicated dietary patterns in pancreatic pathophysiology, but inherent biases, such as reverse causation and unmeasured confounding, obscure true causal relationships^[6]^. For example, the observed link between alcohol consumption and pancreatitis risk might be influenced by other associated behaviors, such as smoking, rather than being a direct result of alcohol itself. Therefore, it is valuable to analyze the complexity of isolating dietary effects from other lifestyle factors. Similarly, the inconsistent findings regarding the impact of fats, fruits, and processed meats on pancreatic health propose the necessity for robust methodologies^[7]^. Such approaches are crucial for accurately assessing risk factors, especially for diseases with long latency periods like pancreatic cancer, where early detection and prevention are key.

Mendelian randomization (MR) circumvents these challenges by employing genetic variants as instrumental variables (IVs) to infer causality, assuming genetic predisposition to dietary habits is less susceptible to confounding^[8]^. MR is a method that uses genetic IVs as proxies to determine the causal effects of modifiable exposures, such as diet, on disease outcomes^[9]^. While MR has clarified causal links between diet and digestive system disease, its application to pancreatic diseases remains nascent^[10]^. Furthermore, prior studies have focused narrowly on single nutrients or lacked granularity in dissecting disease subtypes, leaving gaps in understanding how specific dietary components differentially influence acute, chronic, and malignant pancreatic outcomes^[11,12]^.

Therefore, this study employs a two-sample MR framework to systematically evaluate the causal relationships between 26 dietary exposures and three pancreatic endpoints, utilizing genome-wide association data from the UK Biobank (exposures) and the FinnGen consortium (outcomes). This study has been reported in accordance with the Transparent and Integrated Reporting Guidelines for Mendelian Randomization (TITAN) criteria^[13]^. By integrating rigorous genetic instrument selection, sensitivity analyses, and subtype-specific stratification, we aim to disentangle diet-disease mechanisms while minimizing bias. Our findings advance the evidence base for dietary interventions, offering actionable insights to mitigate the global burden of pancreatic diseases through precision nutrition strategies.

## Materials and methods

### Study overview

**Figure 1.**
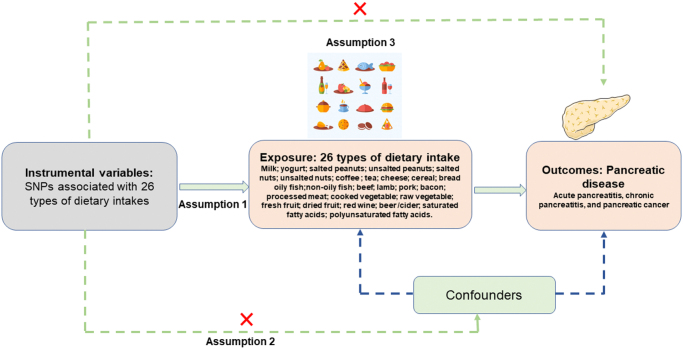
Overview of the study design for MR analysis exploring the causal relationship between dietary intake and pancreatic disease.

We conducted a two-sample MR analysis to elucidate the potential causal relationships between dietary patterns and pancreatic disease outcomes, including acute pancreatitis, chronic pancreatitis, and pancreatic cancer. The study design (Fig. 1) operationalizes MR’s core assumptions through rigorous genetic instrument selection: (1) a strong association between SNPs and specific dietary components; (2) the selected IVs are independent of known confounders; and (3) IV influences results only through dietary pathways. This approach substantially mitigates confounding biases prevalent in traditional nutritional epidemiology.

### GWAS summary data of dietary intake

The genetic association data for dietary exposures were derived from the UK Biobank Genome-Wide Association Study (GWAS) database, a large-scale prospective cohort comprising approximately 500 000 individuals of European ancestry. This dataset provides a robust foundation for MR analyses, given its extensive phenotyping, genetic data quality, and population representativeness. We extracted GWAS summary statistics for 26 distinct dietary factors, including milk, yogurt, salted peanuts, unsalted peanuts, salted nuts, unsalted nuts, bacon, lamb, beer /cider, red wine, coffee, tea, cheese, cereal, bread, oily fish, non-oily fish, beef, pork, processed meat, cooked vegetable, raw vegetable, fresh fruit, dried fruit, saturated fatty acids, and polyunsaturated fatty acids. The data on dietary intake were obtained by asking participants about the frequency of dietary intake in a questionnaire. The relevant questionnaire and the distribution of overall data can be found on the UK Biobank data platform (https://www.ukbiobank.ac.uk).

### GWAS summary data of pancreatic disease

The GWAS summary statistics for pancreatic diseases were derived from the FinnGen database, a nationwide ongoing cohort integrating genetic data with longitudinal electronic health records (EHRs) from Finnish biobanks. Leveraging the R12 release of FinnGen, this analysis focused on three pancreatic phenotypes: acute pancreatitis (8446 cases; 378 749 controls), chronic pancreatitis (4820 cases; 437 418 controls), and pancreatic cancer (3139 cases; 437 418 controls), which were operationalized by using codes of ICD-8, ICD-9, and ICD-10. ICD-10-K85, ICD-9-5770, and ICD-8-5770 were used for diagnosing acute pancreatitis. ICD-10-K86.00, ICD-10-K86.01, ICD-10-K86.08, ICD-10-K86.1, ICD-9-5771, and ICD-8-5771 were used for diagnosing chronic pancreatitis. ICD-10-C25, ICD-9-157, and ICD-8-157 were used for diagnosing pancreatic cancer.

### Genetic IV selection

The IVs were derived from the UK Biobank GWAS database, where original analyses rigorously adjusted for key confounders including age, sex, genetic principal components, study batch, and BMI. Principal component analysis effectively minimizes bias from population structure, while Mendelian randomization’s core assumption further supports IV independence from confounders like socioeconomic status. Detailed covariate adjustments are documented on the website of the UK Biobank – Neale lab (http://www.nealelab.is/uk-biobank/). Genetic IVs were selected following stringent quality control protocols to ensure compliance with MR assumption 1. Initially, single-nucleotide polymorphisms (SNPs) reaching genome-wide significance (*P* < 5 × 10^−8^) in exposure-specific GWAS were prioritized, aligning with established thresholds for robust genetic association. To mitigate biases from linkage disequilibrium (LD), SNPs with pairwise LD *r*^2^ > 0.001 within a 10 000 kb window were pruned using Plink LD clumping. For dietary exposures with limited genetic variants, including milk, yogurt, salted nuts, unsalted nuts, salted peanuts, bacon, lamb, beer, red wine, and unsalted peanuts, a relaxed *P*-value threshold of 1 × 10^−5^ was applied to select IVs. To address weak instrument bias, *F*-statistics were computed using the formula *F* = *R*^2^ × (*N* − 2)/(1 − *R*^2^), where *R*^2^ represents the proportion of exposure variance explained by IVs and *N* is the sample size. IVs with *F* < 10 were excluded.

### Meta-analysis of the estimates from two outcome databases

For results that demonstrated statistical significance in the discovery dataset (FinnGen database), we performed validation using another GWAS meta-analysis dataset, followed by meta-analysis to obtain pooled estimates of each exposure’s effect on pancreatic diseases. We calculated the *I*^2^ statistic to quantify heterogeneity between the estimates from the two studies and assessed their heterogeneity by calculating the *P*-value of Cochran’s *Q* test. When no heterogeneity was observed between the two databases, fixed-effects model meta-analysis was employed to pool estimates for each exposure across both outcome datasets. All meta-analyses were conducted using the “meta” package in R software.

### Multivariable MR analyses

To investigate the direct effects of dietary factors on pancreatic diseases, we performed multivariable Mendelian randomization (MVMR) analysis, an extension of univariable MR that enables simultaneous assessment of causal effects for multiple exposures. The SNPs used in our multivariable MR analysis comprised a combination of IVs for each individual exposure. We restricted our analysis to SNPs within a 10-Mb window at a LD threshold of *r*^2^ < 0.01. The multivariable MR analyses were conducted using the “TwoSampleMR” and “MendelianRandomization” packages in R software.

### Statistical analysis

To explore causal relationships between dietary intake and pancreatic disease, the inverse variance weighted (IVW) method was employed as the primary analytical framework. The IVW method, which aggregates genetic variant–specific effect estimates weighted by their precision, is widely regarded as the gold standard for causal inference in MR when horizontal pleiotropy is absent or negligible. To enhance robustness and address potential violations of MR assumptions, a suite of complementary methods was deployed. Additionally, MR-Egger, weighted median, simple mode, and weighted mode methods were utilized as supplementary analyses. False discovery rate (FDR) was calculated using the Benjamini–Hochberg procedure for multiple comparisons. To enhance robustness and address potential violations of MR assumptions, a suite of complementary methods was deployed. The MR-PRESSO method was used to identify and correct outlier SNPs driving heterogeneity, ensuring that results were not unduly influenced by aberrant genetic effects. Cochrane’s *Q* test was performed to quantify statistical heterogeneity across instruments, with *P* < 0.05 indicating significant heterogeneity. The MR pleiotropy test was conducted to rule out widespread variant effects on unrelated pathways, a critical step in maintaining causal interpretability. All statistical analyses were conducted using R 4.3.0 software with the MRPRESSO, MendelianRandomization, and TwoSampleMR packages.

## Results

### Dietary intake and acute pancreatitis

**Figure 2.**
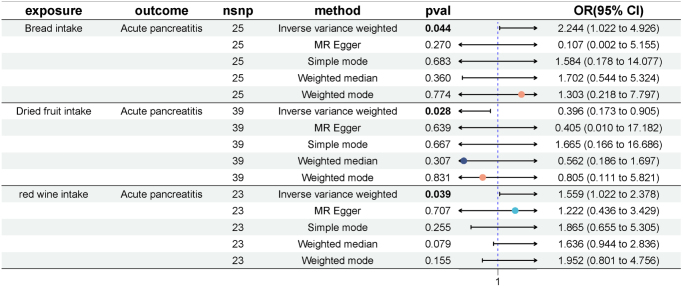
Forest plot of MR results for dietary intake associated with acute pancreatitis. nsnp, nonsynonymous single-nucleotide polymorphism; OR, odds ratio; CI, confidence interval.

Supplemental Digital Content Table S1, available at: http://links.lww.com/JS9/F181 showed the selected SNPs significantly associated with the 26 dietary intakes. All selected SNPs exhibited *F*-statistics well above the conventional threshold of 10 (range: 19.52–700.02), indicating minimal risk of weak instrument bias in the analysis. MR analysis identified three diet-phenotype associations with acute pancreatitis. In IVW models, dried fruit intake emerged as a protective factor for acute pancreatitis (OR = 0.396, 95% CI: 0.173–0.905, *P* = 0.028, FDR = 0.381) (Fig. 2). Conversely, red wine intake (OR = 1.559, 95% CI: 1.022–2.378, *P* = 0.039, FDR = 0.381) and bread consumption (OR = 2.244, 95% CI: 1.022–4.926, *P* = 0.044, FDR = 0.381) were associated with increased risk of acute pancreatitis (Fig. 2). While the *P*-values for these associations were below 0.05, indicating statistical significance, the FDR values exceed this threshold, suggesting that the evidence for these specific dietary influences may require further validation.

### Dietary intake and chronic pancreatitis

**Figure 3.**
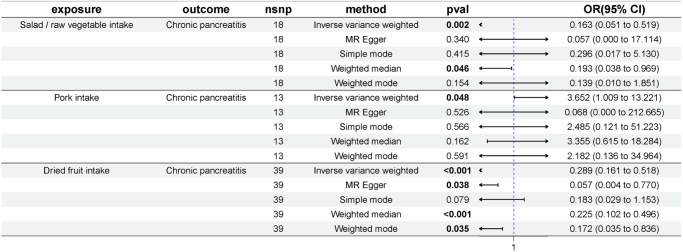
Forest plot of MR results for dietary intake associated with chronic pancreatitis. nsnp, nonsynonymous single-nucleotide polymorphism; OR, odds ratio; CI, confidence interval.

For chronic pancreatitis, MR analysis revealed two protective and one harmful dietary association (Fig. 3). IVW results showed that dried fruit (OR = 0.289, 95% CI: 0.161–0.518, *P* < 0.001, FDR < 0.001) and salad/raw vegetable intake (OR = 0.163, 95% CI: 0.051–0.519, *P* = 0.002, FDR = 0.028) demonstrated protective effects against chronic pancreatitis. In contrast, pork intake (OR = 3.652, 95% CI: 1.009–13.221, *P* = 0.048, FDR = 0.289) emerged as a risk factor for chronic pancreatitis. The low *P*-values and consistent FDR of the protective factors emphasize the robustness of their causal effects, suggesting that the intake of dried fruits and salad/raw vegetables may play a significant role in reducing the risk of chronic pancreatitis.

### Dietary intake and pancreatic cancer

**Figure 4.**
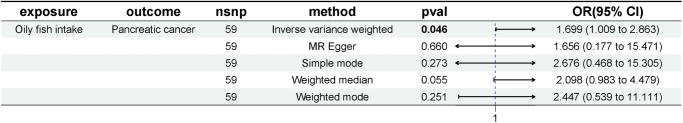
Forest plot of MR results for dietary intake associated with pancreatic cancer. nsnp, nonsynonymous single-nucleotide polymorphism; OR, odds ratio; CI, confidence interval.

The IVW results showed that only one dietary intake was linked with pancreatic cancer. Oily fish intake (OR 1.699, 95% CI 1.009–2.863; *P* = 0.046, FDR = 0.764) showed a significant positive association with pancreatic cancer (Fig. 4). The differing *P*-value and FDR for oily fish intake suggest that its association with pancreatic cancer needs further validation to confirm causality.

### Sensitivity analyses

To validate the robustness of causal relationships between dietary patterns and pancreatic disease, a comprehensive suite of sensitivity analyses was conducted. Cochrane’s *Q* test showed that heterogeneity was not detected in all the positive studies (Supplemental Digital Content Table S2, available at: http://links.lww.com/JS9/F182). Concurrently, the MR-Egger intercept test results revealed no evidence of horizontal pleiotropy, confirming that selected SNPs act primarily through dietary exposure rather than directly influencing pancreatic disease outcomes (Supplemental Digital Content Table S3, available at: http://links.lww.com/JS9/F183). Visual assessments via scatter plots (Supplemental Digital Content Figure S1, available at: http://links.lww.com/JS9/F184) and funnel plots (Supplemental Digital Content Figure S2, available at: http://links.lww.com/JS9/F184) demonstrated symmetric distributions of genetic effect estimates, underscoring the absence of small-study bias or systematic asymmetry in the data. The leave-one-out analysis showed that removing any individual SNP had a negligible impact on effect estimates (Supplemental Digital Content Figure S3, available at: http://links.lww.com/JS9/F184).

### Meta-analysis of the estimates from two outcome databases

**Figure 5.**
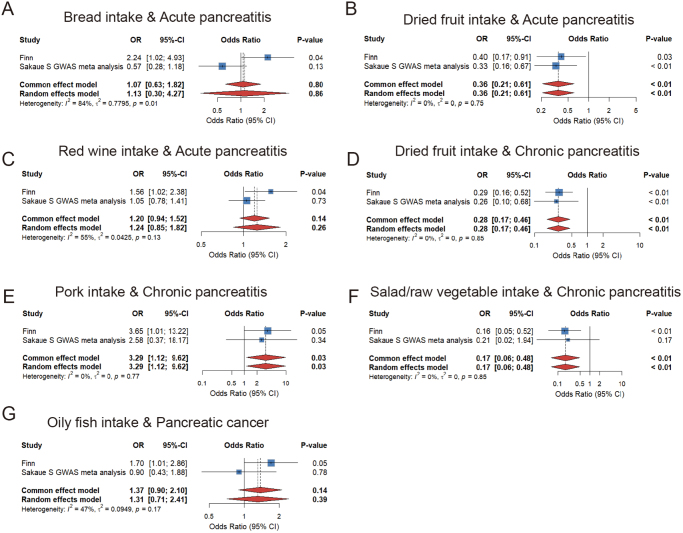
Forest plot of meta-analysis of the estimates from two outcome databases. (A) Bread intake and acute pancreatitis; (B) dried fruit intake and acute pancreatitis; (C) red wine intake and acute pancreatitis; (D) dried fruit intake and chronic pancreatitis; (E) pork intake and chronic pancreatitis; (F) salad/raw vegetable intake and chronic pancreatitis; and (G) oily fish intake and pancreatic cancer.

The meta-analysis revealed no statistically significant association between bread intake or red wine intake and acute pancreatitis. However, dried fruit intake demonstrated a consistent inverse association with acute pancreatitis in both outcome datasets. For chronic pancreatitis, the meta-analysis results of dried fruit, salad/raw vegetable, and pork intake were statistically significant, and were aligned with the findings from the FinnGen database. For pancreatic cancer, oily fish intake did not show a significant positive association with pancreatic cancer in another database, nor in the meta-analysis (Fig. 5).

### Multivariable MR analyses

**Figure 6.**
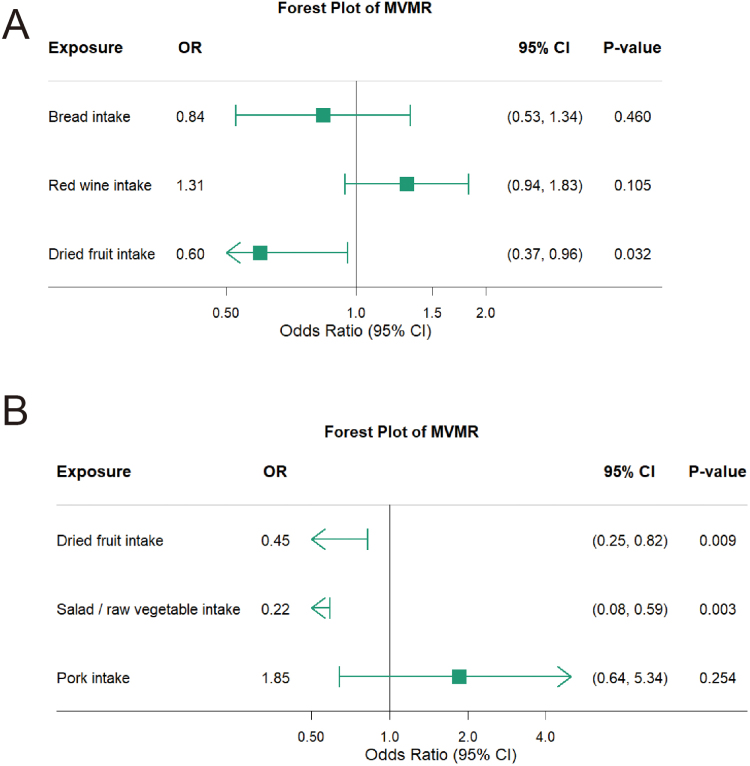
Forest plot of MVMR for multiple dietary phenotypes. (A) Bread, dried fruit, and red wine intake on acute pancreatitis; (B) pork, dried fruit, and salad/raw vegetable intake on chronic pancreatitis.

In order to examine whether the diets have an impact on each other, we conducted MVMR on the foods that affect acute pancreatitis and chronic pancreatitis. The results revealed a significant independent causal relationship between dried fruit intake and acute pancreatitis. In addition, dried fruit and salad/raw vegetable intake showed a significant independent causal relationship with chronic pancreatitis (Fig. 6).

**Figure 7.**
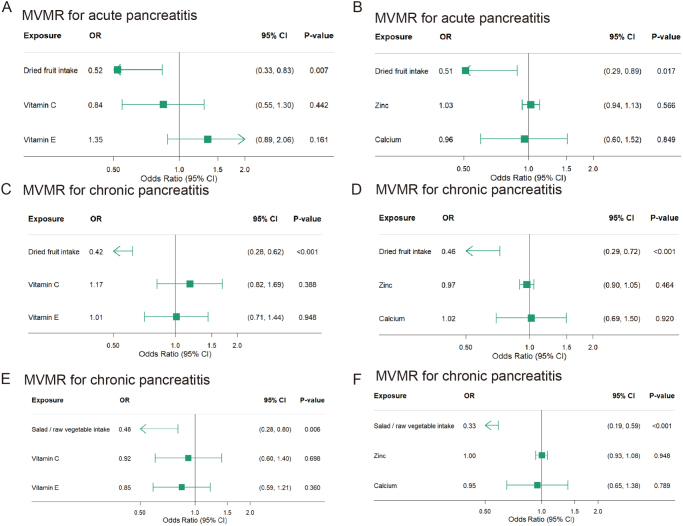
Forest plot of MVMR for trace elements or vitamins. (A) Dried fruit intake, vitamin C, and vitamin E on acute pancreatitis; (B) dried fruit intake, zinc, and calcium on acute pancreatitis; (C) dried fruit intake, vitamin C, and vitamin E on chronic pancreatitis; (D) dried fruit intake, zinc, and calcium on chronic pancreatitis; (E) salad/raw vegetable intake, vitamin C, and vitamin E on chronic pancreatitis; and (F) salad/raw vegetable intake, zinc, and calcium on chronic pancreatitis.

To examine whether diet affects acute pancreatitis and chronic pancreatitis through other factors, trace elements and vitamins were adjusted in MVMR analysis. After adjusting for zinc, calcium, vitamin C, and vitamin E, dried fruit remained as having a significant protective effect on acute pancreatitis and chronic pancreatitis. In addition, salad/raw vegetable intake also showed a significant protective effect on chronic pancreatitis after adjustments (Fig. 7).

## Discussion

Exploring the nexus between dietary patterns and pancreatic health is imperative, given the increasing prevalence of pancreatic disorders and the substantial role that nutrition may play in their etiology and progression^[14]^. Our study used MR to elucidate the causal relationships between dietary intake and three forms of pancreatic disease. Here, we identified significant associations between dried fruit intake and reduced risk of both acute and chronic pancreatitis, increased risk of acute pancreatitis with red wine and bread consumption, and a positive association between oily fish intake and pancreatic cancer. These findings emphasize the potential of dietary modification in the prevention and management of pancreatic diseases, and deserve further discussion on the underlying mechanisms and clinical implications.

Firstly, the protective effect of dried fruit intake against both acute and chronic pancreatitis is a novel finding of our study. Dried fruits, rich in antioxidants and dietary fiber, may contribute to reduced systemic inflammation and oxidative stress, mechanisms known to exacerbate pancreatitis^[15]^. Additionally, specific dried fruits such as prunes and dates, which contain high levels of potassium and magnesium, may particularly benefit pancreatic health by modulating cellular ion balance and reducing inflammatory responses^[16]^. This suggests that incorporating dried fruits into the diet could be a simple, preventative dietary modification to lower pancreatitis risk. Our report is consistent with the study of Bolling *et al*, which highlights the potential anticancer benefits of dried fruits and nuts, noting an inverse relationship between pancreatic cancer risk and improved survival outcomes^[17]^. Furthermore, we also present the protective effect of salad and raw vegetable intake against chronic pancreatitis, suggesting their potential role in reducing pancreatic inflammation. The high levels of vitamins, minerals, and antioxidants in these foods may contribute to their beneficial impact on pancreatic health^[18]^. Further research should aim to dissect the specific components of dried fruits that confer this protective effect, including their role in maintaining pancreatic cell integrity and function.

Secondly, our findings also indicate that red wine and bread consumption are associated with an increased risk of acute pancreatitis. This aligns with existing literature suggesting that certain types of alcohol and high glycemic index foods can exacerbate pancreatic inflammation^[19]^. Notably, the combination of alcohol with high-fat food items, often consumed together with bread, may synergistically increase the risk, potentially due to enhanced oxidative stress and lipid peroxidation in pancreatic cells. The fermentation process in bread and the specific alcohol content in red wine might play roles in these adverse effects. Public health strategies should consider these findings when developing guidelines aimed at reducing acute pancreatitis incidence, emphasizing the importance of moderating the intake of combinations of harmful dietary components.

Additionally, red wine is a complex beverage primarily composed of water and ethyl alcohol, along with various other molecules, such as polyphenols, organic acids, tannins, minerals, vitamins, and biologically active compounds, all of which contribute to its unique flavor and characteristics. Ethanol is a well-known risk factor for acute pancreatitis, and our study shows that red wine containing ethanol is related to increased acute pancreatitis risk^[20]^. The polyphenols in red wine may have a protective effect, which complicates the interpretation of our research results. For instance, epidemiological studies have also shown that moderate consumption of red wine can reduce the risk of cardiovascular disease and diabetes^[21]^. The reasons for the contradictions in the conclusion may lie in the alcohol content, the frequency of drinking, and the differences in organs. In addition, genetic variations in red wine selection may be associated with overall alcohol consumption, which may affect the specificity of the observed association. Although our analysis provides evidence into the relationship between red wine and acute pancreatitis, it is crucial to take into account the broader context of alcohol consumption. Therefore, exploring the respective contributions of different types of alcohol, including red wine, to the risk of acute pancreatitis may involve a more detailed analysis of individual components to distinguish the impact of each component.

Thirdly, the association between oily fish intake and an increased risk of pancreatic cancer is particularly intriguing given the general perception of oily fish as a health-promoting food due to its high omega-3 fatty acid content. This counterintuitive result may reflect complex interactions between fats and tumor biology in the pancreas, or perhaps confounding dietary or genetic factors not fully accounted for in the analysis^[22]^. It is possible that the source and purity of the fish oil, as well as the presence of environmental contaminants such as heavy metals, might be critical in this association. These factors could modulate the effects of omega-3 fatty acids, potentially leading to adverse cellular changes in the pancreas^[23]^. This finding warrants a deeper investigation into the types of fats consumed and their metabolic impacts specific to pancreatic tissue, including a focus on the environmental and processing factors that affect the quality of dietary fats.

While certain dietary elements show a significant correlation with pancreatic health, our research also highlights the existence of dietary components that appear to have a neutral impact on the incidence and progression of pancreatic diseases. For instance, our analysis did not reveal any significant associations between the consumption of dairy products, such as milk and cheese, and the risk of developing either acute or chronic pancreatitis, nor pancreatic cancer. This neutrality could be attributed to several factors. Firstly, the biochemical composition of dairy products, which includes a balance of fats, proteins, and carbohydrates, might not significantly alter pancreatic function or stress. Secondly, the presence of calcium and other vitamins in dairy might help counteract any potential negative effects of other components. Moreover, the impact of such foods may vary among individuals due to genetic differences in metabolism or the presence of other dietary factors that mitigate or exacerbate their effects. This observation confirms the complexity of dietary influences on pancreatic health and suggests that while some foods might be directly linked to disease risk, others may have a more ambiguous or context-dependent role. Further studies are needed to explore these relationships more deeply, particularly through longitudinal dietary assessments and controlled intervention trials.

We also observe that some conclusions from our study may diverge from previous reports, particularly regarding the association between oily fish consumption and cancer risk. These apparent contradictions prompt important considerations. First, the relationship between dietary fats and cancer risk is complex. While omega-3 fatty acids are recognized for their anti-inflammatory effects, the overall impact of oily fish intake could be affected by factors such as the species of fish, cooking methods, and the presence of environmental pollutants, heavy metals, or polychlorinated biphenyls, which might counteract the benefits associated with omega-3 fatty acids^[23]^. Second, despite the goal of MR to reduce confounding, unmeasured confounders may still influence the results. For example, individuals who consume higher amounts of oily fish might also engage in other dietary or lifestyle behaviors that elevate cancer risk, such as increased caloric intake or lower physical activity levels^[24]^. Notably, our supplementary findings indicate that another database and a meta-analysis did not reveal a significant positive correlation between oily fish consumption and pancreatic cancer. This observation further supports our skepticism regarding the link between oily fish intake and pancreatic cancer risk, suggesting the possibility of statistical artifacts or other confounding influences. Lastly, the conclusions we draw are intricately tied to the databases and analytical approaches we utilized, with discrepancies in findings potentially arising from variations in data sources. Do patients benefit from omega-3 fatty acids? These results highlight the necessity of employing a diverse range of data for validation when exploring the relationship between diet and cancer risk.

To enhance the practical applicability of our findings, we contextualized the effect sizes by converting the standard deviation (SD) of dietary intake into tangible consumption amounts. For instance, an increase of 1 SD in nut intake corresponds to approximately 1.82 servings per day, which is associated with an odds ratio (OR) of 0.82 for chronic pancreatitis. This suggests that increasing nut consumption by about two servings daily (roughly equivalent to a handful) could reduce the risk by 18%. This conversion not only clarifies the health implications of dietary choices but also empowers individuals and healthcare professionals to make informed decisions regarding nut consumption. By presenting these concrete interpretations, we aim to bridge the gap between statistical findings and everyday dietary practices, thereby enhancing the relevance of our research in public health discourse. Furthermore, detailed information on the relevant questionnaire and the SD of dietary intake can be found on the UK Biobank data platform (https://www.ukbiobank.ac.uk). This clarification is intended to further improve the transparency and utility of our study.

This study employs MR to explore the causal relationships between diet and pancreatic disease, a topic often complicated by confounding factors in traditional observational research. Our approach involved the systematic application of MR analysis to interrogate 26 dietary components across three pancreatic endpoints. The careful selection of genetic IVs and the use of robust statistical methods contribute to the reliability of our findings. Our results provide insights into how diet influences pancreatic health and suggest that dietary modifications could participate in the prevention and management of pancreatic diseases. This methodological rigor helps clarify potential pathways through which diet affects pancreatic health^[25]^. We also emphasize the need to include diverse populations in genetic research to ensure the generalizability of our findings. By adopting a more measured approach to the analysis of complex genetic and dietary data, our study contributes to a better understanding of how targeted dietary strategies could potentially alleviate the impact of pancreatic diseases.

In our study, the inconsistency between raw *P*-values and FDR-adjusted results raises important questions regarding the coherence of our conclusions. This phenomenon may arise from the effects of multiple testing and the inherent variability among individuals within the sample. While raw *P*-values provide initial insights into specific associations, FDR adjustments offer a more nuanced understanding of significance within the context of multiple comparisons^[26]^. Such discrepancies do not imply that the results are unreliable; rather, they underscore the need for caution in interpreting statistical outcomes. Logically, raw *P*-values reflect the relationship between observed effects and the null hypothesis, whereas FDR adjustments account for the potential risk of false positives introduced by multiple testing^[27]^. Consequently, results with raw *P*-values below 0.05 may appear significant without correction, yet FDR analysis may deem them insufficiently robust when considering the overall testing burden. This situation indicates that while certain dietary factors seem to correlate with pancreatic outcomes in preliminary analyses, a more stringent statistical framework necessitates careful interpretation. Moreover, the observed discrepancies highlight the efficacy of different validation methods. Our study employed various statistical approaches to evaluate the relationships between dietary exposures and pancreatic diseases, thereby enhancing the reliability of our findings. Future research, including independent validation and analyses with larger sample sizes, will be crucial in confirming these associations and improving the consistency of conclusions. Thus, despite the current inconsistencies, our findings continue to contribute valuable insights into the potential role of dietary factors in pancreatic disease, paving the way for further investigation.

Despite the strengths of our study, several limitations still warrant mention. First, the generalizability of our findings may be limited to the populations of European descent, given the demographic characteristics of the UK Biobank and FinnGen databases. Future studies should aim to replicate these findings in more diverse populations, especially in pancreatic disease studies. Secondly, the reliance on self-reported dietary data in our study introduces potential sources of error, such as recall bias and measurement inaccuracies. Participants may not accurately remember or may underreport or overreport their food intake, leading to discrepancies between reported and actual consumption. This methodological limitation can affect the precision of our dietary exposure assessment and subsequently influence the strength and direction of the reported associations. Lastly, while we have minimized potential confounders and used multiple methods to address pleiotropy and heterogeneity, residual confounding cannot be entirely ruled out^[28]^. Further studies using randomized controlled trials or different genetic databases could help validate and extend our findings, especially in the context of pancreatic diseases.

## Conclusion

Our MR analysis identified protective effects of dried fruit and salad/raw vegetables against pancreatic diseases, while red wine, bread, and pork may elevate risk. These findings emphasize the potential of targeted dietary modifications in mitigating the pancreatic disease burden, offering actionable insights for preventive public health strategies. Moreover, these findings call for further investigation of the underlying mechanisms to enhance diet-based preventive strategies.

## Data Availability

All the datasets displayed in this study can be obtained in the article. Further questions can be directed to the corresponding author.
